# Starvation can diversify the population structure and virulence strategies of an environmentally transmitting fish pathogen

**DOI:** 10.1186/1471-2180-14-67

**Published:** 2014-03-14

**Authors:** Lotta-Riina Sundberg, Heidi M T Kunttu, E Tellervo Valtonen

**Affiliations:** 1 Department of Biological and Environmental Science, University of Jyväskylä, Po box 35, FI-40014 Jyväskylä, Finland; 2 Centre of Excellence in Biological Interactions, University of Jyväskylä, Po box 35, FI-40014 Jyväskylä, Finland

**Keywords:** *Flavobacterium columnare*, Starvation, Trade-off, Transmission, Virulence

## Abstract

**Background:**

Generalist bacterial pathogens, with the ability for environmental survival and growth, often face variable conditions during their outside-host period. Abiotic factors (such as nutrient deprivation) act as selection pressures for bacterial characteristics, but their effect on virulence is not entirely understood. “Sit and wait” hypothesis expects that long outside-host survival selects for increased virulence, but maintaining virulence in the absence of hosts is generally expected to be costly if active investments are needed. We analysed how long term starvation influences bacterial population structure and virulence of an environmentally transmitting fish pathogen *Flavobacterium columnare.*

**Results:**

*F. columnare* populations in distilled water and in lake water were monitored for 5 months. During the experiment, the population structure of *F. columnare* diversified by rough and soft colony morphotypes appearing among the ancestral rhizoid ones. After 5 months starvation in lake water, the virulence of the starved and ancestral bacterial isolates was tested. The starved rhizoid isolates had significantly higher virulence than the ancestral rhizoid, whereas the virulence of the rough isolates was low.

**Conclusions:**

We suggest that *F. columnare* population diversification is an adaptation to tolerate unpredictable environment, but may also have other biological significance. Maintaining and increasing virulence ensures efficient invasion into the host especially under circumstances when the host density is low or the outside-host period is long. Changing from rhizoid into a rough morphotype has trade-offs in making bacteria less virulent and unable to exploit the host, but may ensure bacterial survival under unpredictable conditions. Our study gives an example how abiotic selection can diversify virulence of environmentally transmitting bacterial pathogen.

## Background

In the real world the complexity of host-pathogen relationships extends beyond traditional theories, because not all bacterial pathogens suffer from transmission-virulence trade-off [1,2], but are able to survive and reproduce in the environment outside the host. Generalist bacterial pathogens, with the ability for environmental survival and growth, often face variable conditions during their outside-host period. In contrast to obligate pathogens capable for replicating only within the host, these opportunistic pathogens need to adjust their behavior to maximize both the outside-host and within-host survival and growth [3]. However, pathogen evolution is often studied from the within-host perspective, with less emphasis given on opportunists, and how their outside-host phase of the life cycle affects virulence. Revealing environmental factors that cause changes in bacterial behavior and virulence is a key element to understand how disease epidemics emerge, how pathogen dynamics fluctuate, and how the selection pressures in the environment may drive virulence evolution. The dynamics of environmentally growing bacteria are hard to predict, as in many cases their outside-host growth and survival are poorly known.

Pathogenic bacteria with outside-host growth and transmission are subjected to abiotic stress. In aquatic environment nutrient deprivation can be a major selection pressure for bacteria, causing changes in bacterial cell structure and life cycle strategies. Furthermore, abiotic environmental conditions can fluctuate rapidly and increase or decrease probability of host invasion and pathogen virulence. Thus genetic and/or phenotypic diversity is maintained in bacterial populations to ensure survival under unpredictable conditions. The bacteria living in the environment face also other threats: parasitism, predation and competition. Parasitism by bacterial viruses, bacteriophages, is a major driver for diversification of bacterial populations [4-7], similarly to predation by protozoans [8-10]. Environmentally growing bacteria also face competition, that may prevent their ability to invade host populations and cause epidemics [11]. Analyzing the selection pressures that have impact on virulence of opportunistic pathogens is important, because these pathogens can maintain infective populations in the environment that act as sources of disease epidemics.

Ability to survive and grow in the environment gives advantages to a pathogenic bacterium. However, empirical evidence on how environmental selection pressures independently affect bacterial virulence is needed. Generally, pathogens with environmental transmission are expected to have high virulence, especially if infectivity is maintained over long periods [12]. In cases when the host density is low (in nature), long survival increases the probability of successful transmission.

*Flavobacterium columnare* (Bacteroidetes) is the causative agent of columnaris disease in farmed freshwater fish worldwide [13-15]. The source of the disease outbreaks at fish farms are in the ambient waters [16,17], and the bacterium can survive outside the host for long periods whereas the survival in a batch culture decreases rapidly [17-19]. Due to its ability to grow outside the host, *F. columnare* can be considered as an opportunistic pathogen, in contrast to obligate pathogens that can grow only inside the host. As a response to starvation and exposure to lytic phages, *F. columnare* changes colony morphotype [17,19,20]. Loss of the ancestral rhizoid morphotype in a serial culture and in the phage-resistant isolates leads to parallel loss of virulence [19], but the effect of extended starvation on population structure dynamics and virulence of the bacterium is not known. Yet, this knowledge is important as the infective period of *F. columnare* is short in boreal and temperate regions (2–3 warmest months of the year), and correspondingly the outside-host part of the life cycle is long. The aim of this study is to understand the population dynamics of opportunistic bacteria under prolonged starvation (five months) in water, and analyse the consequent effect of starving on bacterial virulence.

## Results and discussion

**Figure 1:**
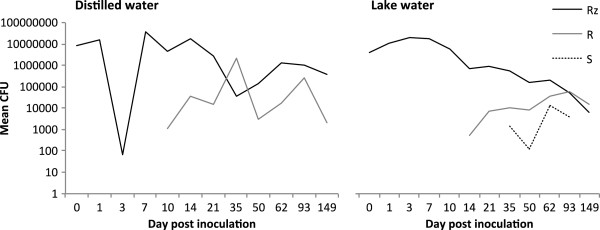
***Flavobacterium columnare *colony morphotype dynamics (as mean colony forming units in log scale) under starvation in distilled water (left) and in lake water (right) during 5 months.** Colony type frequencies were observed by plate counting on days 0, 1, 3, 7, 10, 14, 21, 35, 50, 62, 93, and 149 after start of the experiment. Starvation diversifies the population, as Rough (R) and Soft (S) morphotypes emerge among the ancestral Rhizoid (Rz) colony morphotype.

Survival of *Flavobacterium columnare* under prolonged starvation (5 months) was studied in sterilized lake water and distilled water without any added nutrients. Overall, the bacterium tolerated starvation well, and the population size remained high, from 8.8 × 10^6^ colony forming units (CFU) ml-^1^ in the beginning of the experiment to 3.7 × 10^6^ CFU ml-^1^ in sterile distilled water and to 7.2 × 10^4^ CFU ml-^1^ in sterile lake water at the end of the experiment. The distilled water populations suffered a dramatical drop of population size on day 3 (recovering by day 7). The lake water populations experienced growth during the first week of the experiment, and on days 1, 3, 7 and 10 the average population size was 9.4, 18.3, 15.1 and 4.4 –fold (respectively) when compared to day 0, after which the decrease of population size was constant but slow (Figure 1). The tubes in this experiment were inoculated with the virulent rhizoid morphotype of *F. columnare*, which is typically isolated from diseased fish and nature, while the other (non-virulent) morphotypes have been found to occur after serial culture, starvation or after exposure to lytic phage [17,19,20]. The ancestral rhizoid type remained dominant in distilled water during the 5 months, but frequency of rough morphotype colonies became higher in lake water treatment, increasing to 51% on day 93 and dominating the population (74% of the colonies were rough) at the end of the experiment (Figure 1). The appearance of soft morphotype was observed only in the lake water experiment (in all triplicate tubes).

**Figure 2:**
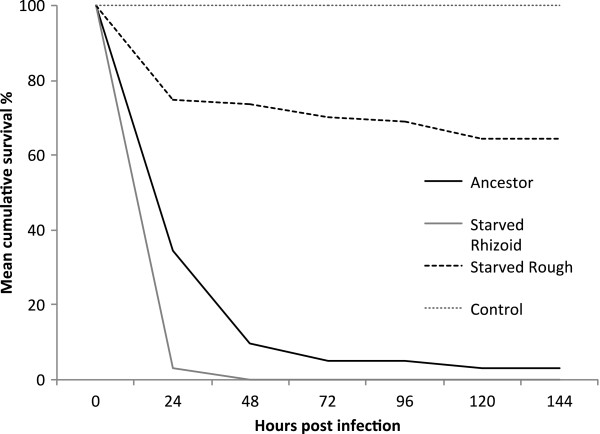
**Mean survival (%) of rainbow trout (*Oncorhynchus mykiss*) after exposure to *Flavobacterium columnare.*** The tested isolates were ancestral Rhizoid (used in inoculation in starvation experiment) and Rhizoid and Rough colony morphotypes isolated after starvation in lake water for 149 days (5 months). Control fish were exposed to sterile culture medium. P < 0.001 for all pairwise comparisons (Table 1).

When compared to the ancestral bacterial strain, the bacteria isolated after 5 months starvation in lake water that maintained their rhizoid colony morphotype had significantly higher virulence than the ancestral rhizoid bacteria (Kaplan Meier survival analysis for whole data *χ*^2^ = 33.89, p < 0.001, pairwise comparisons in Table 1) (Figure 2). The starved isolates from the same lake water treatment that had changed their morphotype to rough had significantly lower virulence than those remaining rhizoid (Table 1, Figure 2).

**Table 1:** **Results of statistical pairwise comparisons (test values *χ***^**2 **^**and p-values) of rainbow trout (*Oncorhynchus mykiss*) populations after exposure to *Flavobacterium columnare *** **(see Figure **2** for survival data)**

	**Ancestor**		**Starved Rhizoid**		**Starved rough**	
	***χ***^**2**^	**p-value**	***χ***^**2**^	**p-value**	***χ***^**2**^	**p-value**
Ancestor						
Starved Rhizoid	33,886	< 0.001				
Starved Rough	92,627	< 0.001	120,889	< 0.001		
Control	227,251	< 0.001	219,356	< 0.001	46,401	< 0.001

Fish were exposed to the ancestral virulent rhizoid isolate and rhizoid and rough isolates isolated after starvation in lake water for 149 days (5 months). Control populations were exposed to sterile culture medium. Kaplan Meier survival analysis for whole data *χ*^2^ = 33.89, p < 0.001.

In the absence of other selective pressures, starvation caused significant diversification in populations maintained in both distilled and lake water treatments. Previously, we have observed similar diversification of colony morphotype in serial cultures [19,20], and during starvation in long-term batch culture as well as in distilled water [17]. This has led us to speculate that the morphotypes may have significance in infection dynamics of the bacterium or for survival under environmental pressures. The present results suggest similarly, that the formation of rough and soft morphotypes may have biological importance for *F. columnare*, but the underlying genetic mechanisms for the colony type variation remains still undetermined.

Under unpredictable conditions it is beneficial for the bacterial population to diversify and be prepared for multiple possible scenarios. The tendency for population diversification is probably strongly favored in nature, thus the phenomenon is often observed also in laboratory. Our data suggest that maintaining the virulent rhizoid colony morphotype during starvation may not be costly under laboratory conditions, but population diversification (the rough and soft morphotypes) may guarantee the persistence of *F. columnare* in the environment. The rhizoid type has higher fitness when the bacterium comes into contact with fish, as it can more efficiently exploit the host as a nutrient source. Although the expression of the rough type may be an efficient strategy to survive under starvation, maintaining the rough morphotype may also have trade-offs in nature. Firstly, the rough type is not able to exploit the fish host, and secondly, the rough types have a slower growth rate and a higher susceptibility for protozoan predation (our unpublished results). In nature and at fish farms this situation is probably solved by a change back to rhizoid type. Indeed, although the morphotype is generally rather stable under laboratory conditions, the morphotype change is reversible [19]. Thus encounter with the host in suitable conditions might cause also the starved rough and soft types *F. columnare* to revert to virulent rhizoid type. The environmental pressures in natural conditions may thus explain why *F. columnare* strains isolated from fish and water in nature are rhizoid in primary culture in the laboratory [17].

Increased virulence of the starved rhizoid type fits to the predictions on higher virulence of pathogens with long-living infective propagules, known as the “sit and wait” strategy [12,21,22]. It is expected that pathogens that have the ability to survive outside the host are not necessarily restricted by the transmission-virulence –trade off, thus their virulence can be high. On the other hand, if contacting a host is a rare occasion, the pathogen must be prepared to invade it, thus the capacity to maintain infectivity even outside the host is important. The relationship between long outside-host survival, environmental transmission and high virulence has indeed been confirmed for several pathogens [23].

*F. columnare* transmission occurs via the environment, as the initial source of bacteria at fish farms are in the intake water [17] and the bacteria shed from the infected and dead fish into the surrounding water [18]. It would be likely that maintaining virulence during outside-host periods would be beneficial especially in nature where fish densities are low, but also in fish farming settings during antibiotic treatment and, more importantly, during cold water season in September-May, when the outbreaks do not occur. However, as the *F. columnare* strains isolated from nature are generally less virulent than those isolated from fish farming [17], the benefits of sit and wait strategy between environmental isolates and disease outbreak isolates needs further studies. While the benefits of the sit and wait strategy would be highest in nature, differences in virulence of the fish farm and environmental isolates may be associated with yet unresolved genetic factors, like horizontal gene transfer, driven by evolutionary conditions in fish farming. Indeed, it has been suggested that high host densities and increased opportunities for transmission in the intensive farming conditions could accelerate pathogen evolution towards higher virulence [24]. Thus fish farm isolates would evolve at a different rate than those isolated from nature. Yet, the role of selection pressures for environmentally transmitting pathogens (e.g. predation, parasitism, competition) that could influence virulence need clarification in both nature and under intensive farming.

In response to starvation, we witnessed population diversification also on virulence. It has been reported that population diversification under parasitism by phages and protozoan predation can cause changes in bacterial virulence either way – for higher or lower virulence [8,25-28]. Understanding factors leading to population diversification and the characteristics of the emerged variants is in the core of infection biology of bacteria also at the molecular level. It has been found that as a response to starvation, *F. columnare* cells may enter viable but not culturable state, in which they remain inactive and may re-emerge when conditions change back to optimal [29]. This has been observed also in other pathogenic bacteria [30,31]. We have no information if *F. columnare* cells in the current study entered non-culturable state, but we have previously observed that the strains grown in batch culture (in Shieh medium) were not culturable after a few weeks, whereas the corresponding inoculations in distilled water survived longer [17].

In addition to being non-culturable, bacterial cells can also respond to starvation by structural changes. *F. columnare* cells have been observed to coil [29], whereas in other bacteria species the cell morphology can change and generally they become shorter, even coccoid [32-35]. The changes in cell surfaces may also cause changes in bacterial colony phenotype [36]. In flavobacterial species, the colony morphology reflects the bacteriums’ ability for gliding motility. The motile bacteria form spreading and rhizoid colonies, whereas the colonies of non-gliding bacteria (rough morphotype) do not spread and have solid edges [19]. The association of motility and virulence has been shown in many bacterial species [37], also in *F. columnare*[19]. The mechanism underlying this association in flavobacteria is most likely the orthology between the gliding motility machinery and the Por-secretion system (PorSS), observed in Bacteroidetes [38]. Thus the loss of virulence in the starved rough type may be caused by structural changes in the gliding motility machinery, which is used in gliding on surfaces and may therefore be energetically demanding to maintain functional in a planktonic growth phase.

In a recent study *F. columnare* strains kept under starvation were reported to suffer from structural changes that recovered after culture in standard media, and the virulence of these bacteria after the two weeks’ starvation decreased, along with their growth rate [29]. These results are in accordance with our results in respect to altered virulence in the outside-host environment. Our current results suggest, however, that the population diversification and the formation of different colony morphotypes under starvation may be a strategy to cope with unpredictable environmental changes [39], while it may also have some other biological functions. *F. columnare* population diversification protects the population from extinction and enables persistence under harsh conditions, similarly to the idea of evolutionary rescue [40].

## Conclusions

Our study gives an example how abiotic selection can diversify virulence of environmentally transmitting bacterial pathogens. Long outside-host survival is in many cases associated with high virulence. Maintaining virulence ensures efficient invasion into the host (sit and wait hypothesis) but may be costly if virulence needs active investments. Here we show that environmentally transmitting bacteria may, indeed, maintain their virulence, but also maintain ability for colony type change to benefit from population diversification, allowing them to be prepared for unpredictable environmental conditions. On the other hand, changing morphotype in *F. columnare* has trade-offs in making bacteria less virulent and thus unable to invade and exploit the host.

## Methods

### Survival in water

The performance of *Flavobacterium columnare* strain C (see [41]) in sterilized distilled and lake water (from Lake Jyväsjärvi, Finland: N 6900494 E 434506) was studied as described in [18]. The bacterium was revived from −80°C glycerol stock (with 10% Fetal Calf Serum) in Shieh medium [42] in room temperature under constant agitation of 100 rpm in an orbital shaker. One ml of fresh, overnight-grown bacterial culture was added to 29 ml of both waters (in triplicate) to reach final bacterial concentration of 8.8 × 10^6^ CFU of bacteria ml^**−**1^ in each of the six tubes. The bacterial count in the beginning of the experiment was determined by plate counting. The tubes were kept in dark at room temperature to ensure stabile condition throughout the experiment.

A 100 μl sample from all tubes was taken for plate counting on days 0, 1, 3, 7, 10, 14, 21, 35, 50, 62, 93, and 149 after start of the experiment. The bacterial counts (as colony forming units) and the morphotypes of the colonies were recorded after 48 h incubation in agarplates at room temperature. On the last day of the experiment, isolates from the lake water experiments were frozen into −80°C for further analyses.

### Virulence experiment

Three individual colonies isolated from the beginning and the end of the experiment (149 days) from lake water treatment were analysed for their virulence. Three rhizoid colonies of ancestral *F. columnare,* 3 colonies of starved rhizoid and 3 starved rough colonies were enriched in Shieh medium overnight. Each culture was used to infect a population of 32–35 rainbow trout fry (mean weight 3.5 g) resulting in a set-up where each colony type (ancestral rhizoid used in inoculation of starvation experiment, and starved rhizoid and starved rough) was tested in triplicate. One population infected with starved rough isolate contained only 16 fish, and three fish populations served as negative controls (total n of rainbow trout in the experiment = 399). The fish were exposed by bathing for 2 h in an aquarium containing 5 × 10^6^ CFU ml^−1^ of *F. columnare* in 7.9 litres of aerated ground water. Water temperature was kept in + 25°C throughout the experiment. Control fish were exposed similarly to sterile Shieh medium. After the challenge, the fish were moved to 15 l experimental flow-through tanks with constant aeration and flow of fresh water. Disease signs and fish mortality were monitored for 6 days. Morbid fish that had lost their natural swimming buoyancy, and did not respond to external stimuli were considered dead and removed from the experiment. They were given terminal anesthetic and killed by cutting the spinal cord, to meet the ethical endpoint of the experiment and to avoid suffering of the fish. The experiment was conducted according to the Finnish Act on the Use of Animals for Experimental Purposes, under permission granted by the National Animal Experiment Board at the Regional State Administrative Agency for Southern Finland (permission number LSLH-2005-10649/ym-23).

### Data analysis

The fish mortality data were analysed with Kaplan-Meier survival analysis using IBM SPSS Statistics 20.

## Competing interests

The authors declare that they have no competing interests.

## Authors’ contributions

L-RS participated in designing the study, performing the experiments and data collection, analysed the data and wrote the manuscript. HK participated in designing the study, performing the experiments and data collection, and helped to draft the manuscript. ETV participated in designing the study, and helped to draft the manuscript. All authors read and approved the final manuscript.
